# Assessment of Polymer Concrete Sample Geometry Effect on Ultrasonic Wave Velocity and Spectral Characteristics

**DOI:** 10.3390/ma14237200

**Published:** 2021-11-25

**Authors:** Kamil Zalegowski

**Affiliations:** Department of Building Materials Engineering, Faculty of Civil Engineering, Warsaw University of Technology, Al. Armii Ludowej 16, 00-637 Warsaw, Poland; k.zalegowski@il.pw.edu.pl

**Keywords:** polymer concrete, ultrasonics, upv, sample shape effect, sample size effect, Fourier-based synchrosqueezing transform

## Abstract

In this paper an analysis of the influence of polymer concrete sample shape and dimensions on ultrasonic wave propagation is carried out. Compositions of tested fly ash polymer concretes were determined using a material optimization approach. The tests were carried out on the samples of three shapes: cubes, beams, and plates. The ultrasonic testing was done by a direct method (transmission method) using a digital ultrasonic flow detector and piezoelectric transducers of 100 kHz central frequency. Propagation of the ultrasonic wave was characterized by pulse velocity. Frequency spectra and time-frequency spectrograms obtained using Fourier transform and Fourier-based synchrosqueezing transform were also presented. The correlation analysis showed that neither the path length nor the lateral dimension to the direction of wave propagation are not statistically significant for the UPV variability. However, a general trend of decrease in the UPV with increasing the path length was noticed. The analysis of the signal in time-frequency domain seemed to be useful in the analysis of particulate composites properties, especially when UPV changes are not clear enough, since it revealed greater differences in relation to changes in sample geometry than frequency spectra analysis.

## 1. Introduction

Polymer concrete (PC) belongs to the group of building particulate composites (i.e., concrete-like composites), where a cement paste was totally replaced with a resin binder [1]. It is obtained by mixing the synthetic resins, pre-polymers or monomers with suitably selected aggregate, followed by hardening of the resin binder. The PC binders are usually two-component sets, the setting and hardening of which is the result of the reaction between resin and hardener. PC may contain a binder hardened by polycondensation (e.g., phenol-formaldehyde resins), accompanied by release of the by-product (e.g., water), or by polymerization-without by-products (e.g., polyester or epoxy resin). Among others, the characteristic properties of PC are high compressive strength, very good chemical resistance, good adhesion to various materials and short time to exploitation [1]. The main fields of PC applications are [2]:

− repair maintenance and anti-corrosion protection (protective and decorative coatings) of building structures;
− flooring, especially industrial floors, as well as floors in hospitals, schools, indoor and outdoor sports facilities and other public utility structures;
− precast elements—acid tanks, drains, manholes, pipes, slabs, highway median barriers, etc.

The usefulness and durability of PC composites depend on the selection of the material composition for obtaining composites with controllable properties [3,4]. However, during service live PC composites are exposed to harmful atmospheric, chemical, and biological factors, which may cause gradual degradation and damage to the material. Therefore, the systematic assessment of durability and diagnostics of PC elements is an important issue. It could be done using non-destructive testing (NDT), which contrary to destructive methods, allows obtaining information on the material properties without deteriorating its microstructure and serviceability. Ultrasonic methods are among others the most common non-destructive techniques used in material science and industry [5]. They are well-known and standardized for traditional building materials, like metals, cement concretes and rocks [6,7,8,9].

One of the oldest and simplest is ultrasonic pulse velocity (UPV) testing. It is commonly used to assess cement concrete homogeneity [10,11,12], compressive strength and monitor its changes over time [13,14,15,16]. The UPV method could be also used to determine material constants (e.g., modulus of elasticity [17]) or detect defects such as voids, delamination or cracks [18,19,20,21]. The usefulness of the UPV method in testing of polymer or polymer–cement composites has been demonstrated too [21,22,23,24,25,26].

The UPV method uses longitudinal elastic waves that are vibrations with a frequency above 20 kHz, which is the upper limit of the audible frequency for humans. The vibrations of higher frequencies are used to perform more accurate measurements, but at the same time the attenuation of ultrasonic pulses is greater, so their energy is lower. Therefore, the ultrasonic testing of heterogenous concrete composites should be carried out using sound waves with the highest possible frequencies, that allow registering good quality waveform after signal propagation in a medium. During the movement, longitudinal waves cause medium particles to vibrate parallel to the direction of wave travel. This type of vibration can travel through solids, liquids, or gases.

**Figure 1:**
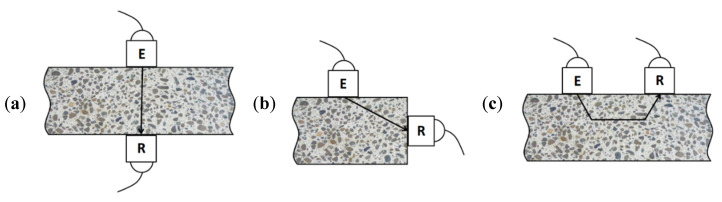
Arrangement of transducers in ultrasonic pulse velocity (UPV) method (based on [10]): (**a**) direct method, (**b**) semi-direct method and (**c**) indirect method. Explanations: E—emitter, R—receiver.

The goal of the UPV test is to determine the travel time, of the ultrasonic wave after its propagation through the tested medium on the known path length (Figure 1). The equipment consists of ultrasonic defectoscope and two piezoelectric transducers, i.e., transmitter and receiver. In most cases, both transducers are located coaxially on the opposite sides of the tested element (Figure 1a) because in this test system the maximum energy of ultrasonic pulse is transmitted and received. Therefore, the direct transmission method is the most sensitive and reliable [27,28]. Whenever two opposite faces are not available, then the transducers could be placed either on perpendicular surfaces (semi-direct method, Figure 1b) or on the same side of the tested member (indirect or surface method, Figure 1c). Indirect transmission is used when only one face of the concrete structure is accessible since it is described as the least accurate testing arrangement. Furthermore, it is stated that measurements on the surface are indicative only of properties of the layers that are close to the surface [27].

Pulse velocity test is relatively easy to carry out, but it is very essential that the test should be performed properly to ensure the reproducibility of UPV readings and that they are dependent only on the properties of the tested concrete rather than by other factors. Thus, it is important to better know and understand factors influencing UPV measurements in building particulate composites. Considering the similarity of the microstructure features of both cement concrete and polymer concrete, it seems reasonable to implement experience gathered using ultrasonic techniques on cement composites into polymer ones. However, it should be remembered that the differences between properties (especially elastic properties) of cement and resin binder, may influence the ultrasonic wave propagation in PC. Generally, ultrasonic wave transmission depends on concrete composition and microstructure, so in the case of PCs it is dependent on the type and content of binder and filler, grain-size distribution of the filler, microfiller type and content, and porosity [2]. It is also necessary to provide adequate adhesion between resin binder and aggregate. There are other factors which are not associated with material composition and microstructure and may also influence UPV in PC composites similarly to cement composites. They are as follows: coupling between the transducers and concrete surface [29,30], presence of steel reinforcement [31,32], concrete temperature and moisture [33,34], and sample dimensions (including path length) and shape [34,35,36,37]. The relation between UPV and sample shape/dimensions seems to be especially interesting, cause ultrasonic method is commonly used in compressive strength estimation of particulate composites [15,38,39,40,41], and it is well known that compressive strength results are strongly dependent on the sample geometry [40,42].

The aim of this paper was to analyze how the shape and dimensions of PC samples may influence the ultrasonic pulse velocity and ultrasonic signal in frequency and time-frequency (TF) domain.

## 2. An Influence of Sample Shape and Dimensions on the Ultrasonic Pulse Velocity (UPV) and Frequency Spectrum of the Ultrasonic Signal

At the beginning it should be mentioned that very limited information is available about sample’s shape and dimensions effect on the ultrasonic pulse velocity in particulate composites, especially in the case of polymer composites. However, generally the pulse velocity of longitudinal stress waves in concrete is related to its elastic properties and density according to following relation (1):

(1) $$V=\sqrt{\frac{E\left(1-v\right)}{\rho \left(1+v\right)\left(1-2v\right)}}$$

where: *E*—dynamic modulus of elasticity, *v*—dynamic Poisson’s ratio, *ρ*—density. Theoretically, the UPV should not be affected by the path length travelled by the wave. Although, in practice, smaller path lengths tend to give more reliable variable and slightly higher pulse velocity because of the inhomogeneous nature of concrete [34]. The ultrasonic wave travelling through a medium is attenuated due to energy scattering on interfaces with phases having distinct properties. Such phases in concrete could be not just voids, cracks, and reinforcement, but aggregate grains too. As a result, the longer is the path length, the greater the ultrasonic waves attenuation. The magnitude of this phenomenon would be more intense if the wavelength of the ultrasonic wave is comparable or smaller than the size of the scatterer. In contrast, RILEM guidelines [43] state that the path length should be long enough to avoid the effect of inhomogeneous nature of concrete on the UPV results. It is suggested that greater path lengths are favorable, since the recommended minimal path length for ultrasonic waves is 100 mm or 150 mm for the concrete with maximum grain size of 30 mm or 45 mm, respectively.

According to ASTM C597 standard 7] the UPV in concrete is independent of the dimensions of the test object provided if waves reflected from boundaries do not complicate the determination of the arrival time of the directly transmitted pulse. This could be achieved if the least dimension of the test object exceeds the wavelength of the ultrasonic vibrations. ASTM C597 guidelines do not specify the impact of aggregate grains size. According to Wiciak P. et al. [35] the minimal sample dimensions specified by ASTM C597 are even insufficient. They recommend that the length of test sample should exceed more than one wavelength recommended by ASTM C597. They investigated an effect of height (path length in the range about 46 mm to 280 mm) and diameter (100 mm, 200 mm, and 300 mm) of cylindrical cement mortar samples on signal and spectra characteristics. The samples were tested with 54 kHz and 850 kHz resonant frequency transducers. The outcome of the research is that for low frequencies the separation between P (longitudinal wave) and S-wave (shear wave) is impossible for short specimens, even if the condition indicated in ASTM C597 is fulfilled. The P-wave is not seen in the signal because of reflections from the side. This made the UPV determination difficult or even impossible.

Stawiski B. et al. [7] also states that the ultrasonic velocities determined on various paths lengths (i.e., height of the samples equals to 4, 8, 16 and 32 cm) for cylindrical samples made of cement concrete differ considerably from each other. The UPV increased with the increase in path length. However, this effect could be more likely caused by changes of lateral dimension, that is sample diameter, which was equal to sample height in this study. In other words, it could be concluded that the smaller the sample, the lower the UPV in concrete. The greatest difference in UPV that authors noticed was between the results obtained for cylinders of 4 cm and 8 cm in diameter (difference in UPV about 6.8%), while the difference in UPV measured on cylinders of Ø8 cm and Ø32 was almost three times lower (difference in UPV about 2.5%). Such a small range of the UPV changes could be considered a consequence of concrete inhomogeneity. Stawiski B. et al. used transducers with resonant frequency 40 kHz, so assuming the UPV in concrete is 3500 to 5000 m/s, the wavelength ranges from 8.8 cm to 12.5 cm. According to ASTM 597 guidelines the minimal sample dimensions in the research of Stawiski B. et al. should exceed 8.8 cm or 12.5 cm depending on the UPV in the tested concrete. This may be an explanation of the character of changes of the UPV in relation to the sample dimensions/path length.

Unlike the rest, Ersoy H. et al. [37] reported that UPV in cement concrete is not affected by path length because the differences in UPV values obtained from different lengths (heights of cylindrical samples) were below 1.5%. Moreover, ultrasonic tests performed on the concrete samples of different diameters (lateral dimension) revealed the differences in UPV values about 1.8%. Based on these results, the authors claimed that UPV is related to the material homogeneity rather than the path length or lateral dimension.

Similar conclusions were drawn by Mantrala S.K. and Vipulanandan C. [44], who investigated the effect of shape and size on the pulse velocity in polymer concrete in form of cylinders and prisms (details given in Table 1). The UPV was measured at frequencies of 50 and 150 kHz. The outcome of the study is that the UPV is independent of sample shape and sizes, regardless of frequency used.

An influence of sample shape on the UPV in cement concrete, among others, was investigated by Sasanispur H. et al. [45] and Godinho J.P. et al. [46]. They used 54 kHz transducers and met the condition set in ASTM 597 regarding the least dimension of the test sample in relation to the ultrasonic wavelength. An outcome of both studies is similar, the shape of the sample generally had no influence on the results. Of course, differences in UPV between samples of cylindrical and cubic geometries were noticed, but in most cases they were negligible and could be due to the concrete inhomogeneity.

Only one paper concerning changes in the spectra of the signals propagated was found, and it only covers the cement concretes. According to Wiciak P. et al. [35] the central frequency of the 54 kHz peak is shifted to the lower frequencies as the length of specimen and diameter rises. On the other hand, it was noticed that increasing sample dimensions narrows the area of the peak associated with the P-wave registered using 850 kHz. This might be the result of smaller influence of side reflections, cause the wavelength is almost 20 times shorter than in the case of 54 kHz transducers. The authors also paid the attention to the effect of attenuation with increasing length of the specimen, especially strong for higher frequency signals.

The transformation of signal in the time domain into the frequency or time-frequency domain may be used to apply signal processing techniques such as the filtering or to extract useful information about propagation medium. Polymer composites are useful materials for repair and protection of building structures, and for manufacturing precast elements. In both cases, there is the need for quality control and diagnosis after application/production and during structural service as well. The PCs are used for production of different precast elements of different shapes, and layers or protective coatings. Advanced signal analysis may be also necessary for instance in testing polymer concretes and concrete like composites modified with waste materials e.g., fly ash, which may significantly influence the concrete properties [24,45,47] and thus propagation of ultrasonic waves in concrete. Therefore, there is an important need to develop non-destructive assessment methods for polymer composites. That is why, in this paper the influence of PC sample geometry on the frequency spectra and time-frequency spectrograms characteristic was investigated alongside the influence of PC sample geometry on the UPV.

## 3. Materials and Methods

In this work an analysis of the influence of polymer concrete sample geometry on the ultrasonic wave propagation is carried out. Polymer concretes used were cementless composites in which synthetic resin was used as the sole binder, and the aggregate was basically the same as for the ordinary concretes, prepared in the form of a blend of basic aggregate fractions, and the so-called microfiller—very fine powder obtained by grinding sand).

**Figure 2:**
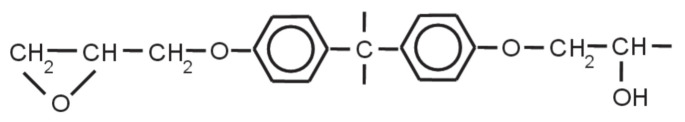
The chemical formula of epoxy resin.

The polymer used to prepare concretes in the study was commercially available epoxy resin Epidian 601 with hardener Z1 (Figure 2, Table 2). The resin used is light yellow, low viscosity epoxy resin modified with a reactive diluent. It is intended for preparation of composites with fillers, floorings, and epoxy–glass laminates, and impregnation of concrete.

The fillers included quartz aggregate of a fraction 0/2 mm and 2/4 mm and quartz powder with maximal particle size about 70 µm as the microfiller. The reason for use of only fine aggregate in this study is that the polymer concretes are often applied in the thin section elements, so due to small thickness of the element/layer, the aggregate of small grain size is needed. In addition, the aggregate was dried to avoid the potential negative effect of moisture on the setting process of the epoxy binder. The proportions between the individual aggregate fractions were determined based on the method of successive approximations, which allowed us to obtain a blend of aggregate with maximum density and minimum cavity.

The proportions between the polymer binder and the aggregate as well as between the polymer binder and the microfiller were the basis of the PC design. In the tested composites (in accordance with the recommendations of Czarnecki L. [1]) the ratio between aggregate and binder (A/B) determined on the basis of specific density, bulk density and cavity of aggregate was 8. The ratio between binder and microfiller (B/M) after considering the resin viscosity and aggregate granulation was determined as 0.4. This is in line with the range 0.4–0.6 recommended by Czarnecki L. [1], where the value 0.4 is taken for resins with low viscosity (*n* ≤ 1.0 Pa·s) and microfillers with diameters greater than 40 µm, and the value of 0.6 is taken in the case of higher viscosity resins (*n* ≥ 2.5 Pa·s) and microfiller with diameters not greater than 30 µm. The other mass ratios of ingredients were sand to aggregate ratio (S/A) and microfiller to aggregate (M/A), which was equal to 0.41 and 0.18, respectively.

The polymer concrete mix preparation process was as follows. First, the binder was prepared by mixing the epoxy resin with the polyamide hardener. Then, a quartz microfiller is added to the binder to create the so-called micromortar. The micromortar is then added to the base aggregate mix. Mechanical mixing made it possible to obtain a sticky, plastic mass, which was then placed in the molds previously covered with an anti-adhesion agent. The mix was placed in 2–3 layers in molds and each time the mix was compacted in the molds on a vibrating stand (vibration time about 5–10 s) in order to deaerate the mix. Samples tested in the presented research were of three shapes: cubes, beams, and plates with different dimensions specified in Table 3. It is also worth noting that the minimal dimension of the prepared samples was equal to 40 mm and was comparable with the ultrasonic wavelength used. After 24 h, the samples were demolded and then stored in laboratory conditions for 13 days. Fourteen days after production, the samples were tested. The resin concretes do not require special curing, at temperature excessing 15 °C they usually reach 80% of the maximum strength [1].

**Figure 3:**
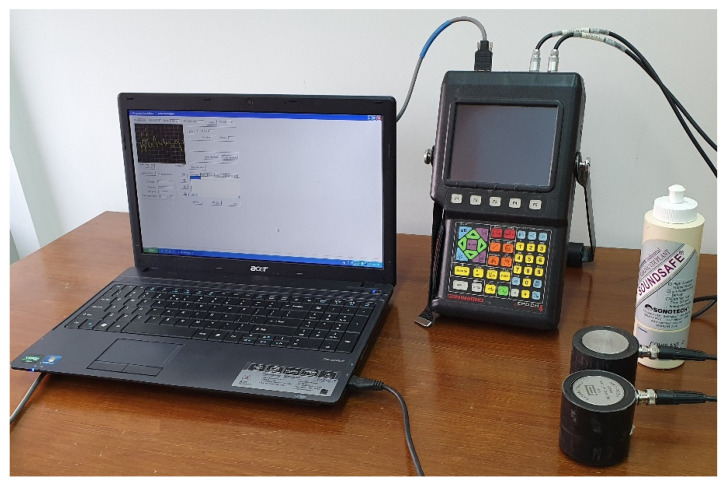
The ultrasonic experimental setup used in the study–ultrasonic defectoscope Epoch4, piezoelectric transducers of 100 kHz central frequency and coupling gel (Panametrics NDT-Olympus NDT, Shinjuku, Tokyo, Japan).

**Figure 4:**
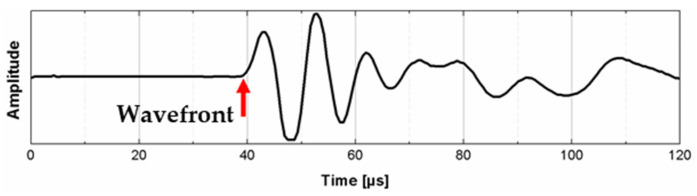
The presentation of registered ultrasonic signal with the indication of the wavefront used to determine the time of wave propagation.

The ultrasonic measurements of samples with different geometries included the determination of ultrasonic pulse velocity, and presentation of the signal in the frequency and time-frequency domains. The ultrasonic testing was done by direct method (transmission method) using a digital ultrasonic flow detector and piezoelectric transducers of 100 kHz central frequency (Figure 3). Firstly, calibration of the ultrasonic equipment was performed using steel calibration standard probes with different heights and known ultrasonic pulse velocities. Calibration consisted of determination of the zero time, which is the time taken from the impulse generation to its introduction in the tested material. This time was used to compute the time of wave propagation on the known distance between transducers. To ensure adequate acoustic coupling between the concrete surface and the special head, commercial coupling gel was applied. The signals were registered using a specialized program and to reduce random error each signal was averaged 10 times. In order to compute UPV, graphs presenting the amplitude changes in the function of time were prepared, and the propagation time of ultrasonic impulse between emitter and receiver was determined on the basis of the position of the wavefront (Figure 4). The wave velocity was computed by dividing the distance between transducers (path length) by the time of wave propagation. The UPV values were determined three times per sample (one value per sample plane) and including the fact that three samples of each size were prepared, finally nine UPV results per sample geometry were computed. To determine whether a sample shape or dimensions (wave path length, lateral dimension to direction of wave propagation) influence the ultrasonic pulse velocity, correlation analysis was used.

An impact of a sample geometry on characteristics of ultrasonic signal in frequency and time-frequency domain was investigated too. In this purpose, fast Fourier transform (FFT) and Fourier-based synchrosqueezing transform (FSST) of ultrasonic signal was performed using Matlab software.

The fast Fourier transforms [49] are optimized algorithms that computes the discrete Fourier transform (DFT) of a sequence, or its inverse (IDFT). The purpose of DFT is to decompose a sequence of values into components of different frequencies. The basic definition of the DFT is (2) and (3):

(2) $$C\left(k\right)=\overset{N-1}{\underset{n=0}{\sum }}x\left(n\right)\;\;\;\;\;W_{N}^{nk}$$

where *n*, *k*, and *N* are integers, $j=\sqrt{-1}$, the basis functions are the *N* roots of unity,

(3) $$W_{N}=e^{-j2\pi /N}$$

and *k* = 0, 1, 2, …, N−1. It could be seen that if the N values of the transform are calculated from the *N* values of the data *x*(*n*), *N^2^* complex multiplications and about the same number of complex additions are required. In practice, despite its usefulness in numerous fields, the DFT is to complex and computing it is often too slow. These challenges could be reduced using one of the most important algorithms in signal processing and data analysis–the FFTs. Popular algorithms use index mapping, to change the one-dimensional DFT into a two- or more dimensional DFT; these for instance include the Cooley–Tukey or Winograd algorithms.

The FFTs are virtually optimized algorithms for the implementation of the DFT. They sample the signal over a time interval and divide it into frequency components, which are single sinusoidal oscillations of different frequencies, characterized by its own amplitude and phase. The FFTs are the basis of frequency domain analysis (spectral analysis), signal filtering, spectral estimation, data compression, and others.

Time-frequency analysis is employed in many engineering fields, such as communication, sonar as a useful tool for analyzing time-varying non-stationary signals. TF analysis is particularly useful for signals composed of many oscillatory components with slowly time-varying amplitudes and instantaneous frequencies. The most typical TF analysis includes short-time Fourier transform (STFT), continuous wavelet transform (CWT), and WignerVille distribution [50,51].

The synchrosqueezing transform [52,53] is generally a kind of reassignment method that aims to sharpen a timescale representation, while remaining invertible. The main idea of reassignment methods is to analyze the local behavior in the time-frequency plane of portions of the representation and determine nearby possible time-frequency concentration candidates which explain it in the best way. Then, each small portion is moved to its “right” place in the time-frequency plane to get more concentrated time-frequency representation, that should give a compatible and precise rendering of the time-frequency properties of the signal. The Fourier-based synchrosqueezed transform is based on the STFT. The STFT of $x\left(t\right)\in L_{2}\left(R\right)$ with a window function $g\left(t\right)\in L_{2}\left(R\right)$ is defined by (4) [54]:

(4) $$V_{x}\left(t,\;\eta \right)=\int_{-\infty }^{\infty }x\left(\tau \right)g\left(\tau -t\right)e^{-i2\pi \eta \left(\tau -t\right)}d\tau =\int_{-\infty }^{\infty }x\left(t+\tau \right)g\left(\tau \right)e^{-i2\pi \eta \tau }d\tau$$

where *t* and *η* is the time variable and the frequency variable, respectively. STFT can be also written as (5) [54]:

(5) $$V_{x}\left(t,\;\eta \right)=\int_{-\infty }^{\infty }\hat{x}\left(\xi \right)\hat{g}\left(\eta -\xi \right)e^{i2\pi t\xi }d\xi$$

were for a signal x(t), its STFT $\hat{x}\left(\xi \right)$ is defined by (6) [54]:

(6) $$\hat{x}\left(\xi \right)=\int_{-\infty }^{\infty }x\left(t\right)e^{-i2\pi \xi t}dt$$

In the STFT based FSST the frequency variable is reassigned, for a signal x(t), at (t, η) for which $V_{x}\left(t,\;\eta \right)\neq 0$, according to (7) [54]:

(7) $$\omega_{x}\left(t,\;\eta \right)=\frac{\frac{\partial }{\partial t}V_{x}\left(t,\;\eta \right)\;}{2\pi iV_{x}\left(t,\;\eta \right)\;}$$

The FSST reassigns the *η* variable by transforming the STFT $V_{x}\left(t,\;\eta \right)$ of x(t) to the quantity $R_{x}\left(t,\;\eta \right)$, on the time-frequency plane (8) [54]:

(8) $$R_{x}\left(t,\;\xi \right)=\int_{\left\{Ϛ:V_{x\left(t,\;\;Ϛ\right)\neq 0}\right\}}V_{x}\left(t,\;Ϛ\right)\delta \left(\omega_{x}\left(t,\;Ϛ\right)-\xi \right)dϚ$$

where *ξ* is the frequency variable.

The FSST can be used to extract the instantaneous frequency and reconstruct the constitutional oscillatory components of a signal in the presence of noise, which may effectively improve the readability of the time frequency representation of non-stationary signals composed of multiple components.

## 4. Results and Discussion

### 4.1. An Effect of Sample Shape and Dimensions on the Ultrasonic UPV

**Figure 5:**
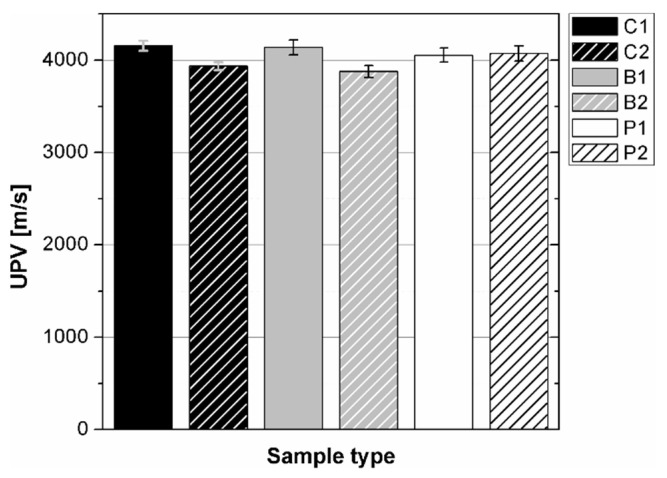
Pulse velocity in epoxy concrete samples of different types.

**Figure 6:**
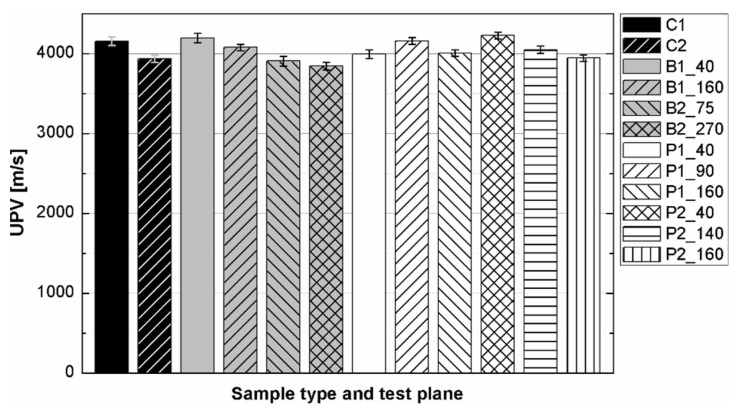
Pulse velocity in epoxy concrete depending on the sample type and test plane.

**Figure 7:**
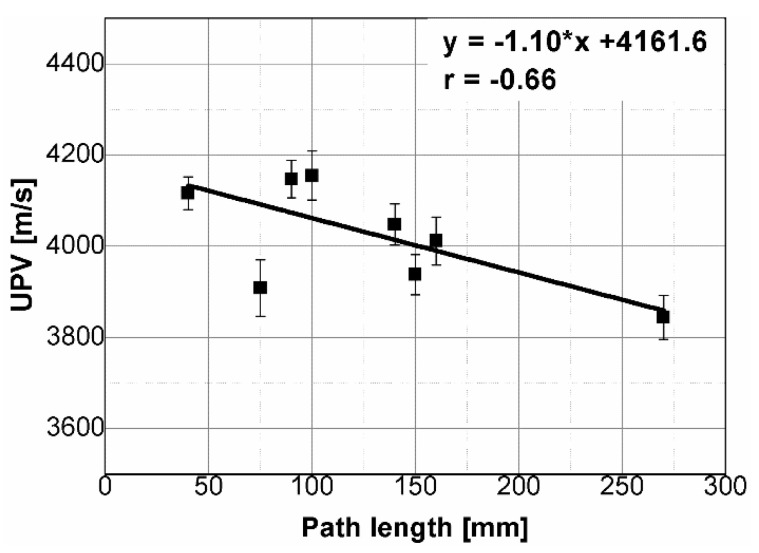
Pulse velocity plotted against path length.

**Figure 8:**
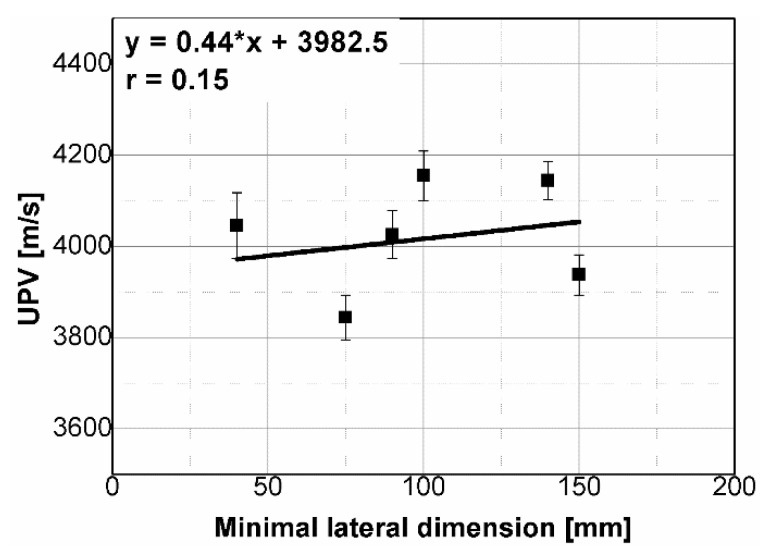
Pulse velocity plotted against minimal lateral dimension.

The results of the ultrasonic tests are presented in Table 4 and on Figure 5, Figure 6, Figure 7 and Figure 8. In general, ultrasonic pulse velocity was very similar, with a ratio of 1 ± 0.03 regardless of whether the comparison involved changes of the UPV with respect to the sample shape or dimensions; these included, first, parallel dimension to direction of wave propagation (path length) and, second, minimal lateral dimension to direction of wave propagation (Table 4, Figure 5 and Figure 6). The above indicates that the changes of the UPV were caused by the inhomogeneous nature of particulate composites, than by the sample geometry. The variability of results obtained for whole samples and for individual test planes was not greater than 2%, and it confirmed that except for the heterogeneity typical for particulate composites, no additional heterogeneities were introduced.

Statistical analysis showed that UPV is correlated with path length with coefficient |*r*| equal 0.66 (Figure 7). That is why, it may be concluded that UPV-path length relation is not statistically significant, but general trend of decrease in the UPV with increasing the path length might be indicated. The correlation of UPV and lateral dimension showed very low |*r*| value of 0.15, thus excluding any kind of relation between these parameters (Figure 8). This is in agreement with Ersoy H. et al. [37], who reported that UPV in cement particulate composites is not affected by path length because the differences in UPV values obtained from different lengths were below 1.5%. Similar conclusions were drawn by Mantrala S.K. and Vipulanandan C. [44] in case of polyester polymer concrete. They investigated the effect of shape and size on the pulse velocity in PC in form of cylinders and prisms, with transducers of 50 kHz and 150 kHz central frequency. The outcome of the study was that the UPV is independent of sample shape and sizes, regardless frequency used. However, it should be mentioned that there are also authors who received different results (in case of cement concretes).

### 4.2. An Effect of Sample Shape and Dimensions on the Spectra Characteristics

**Figure 9:**
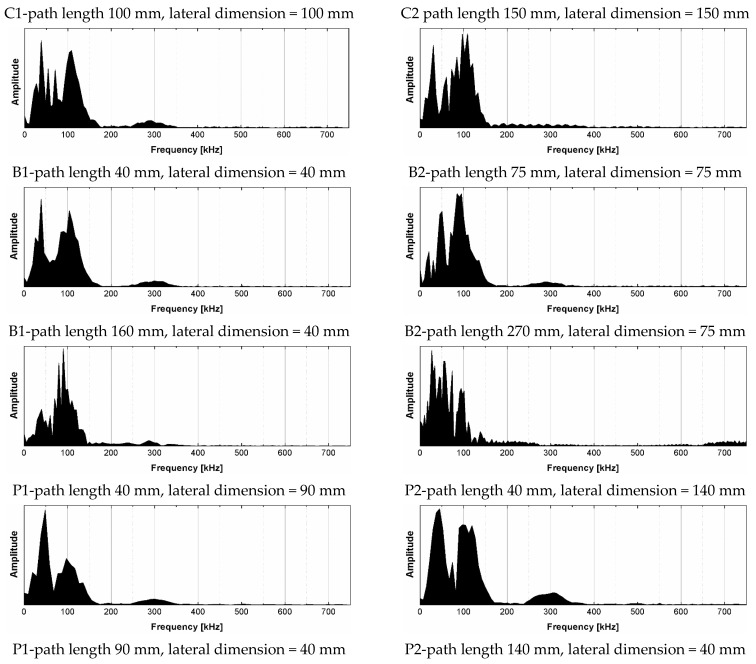

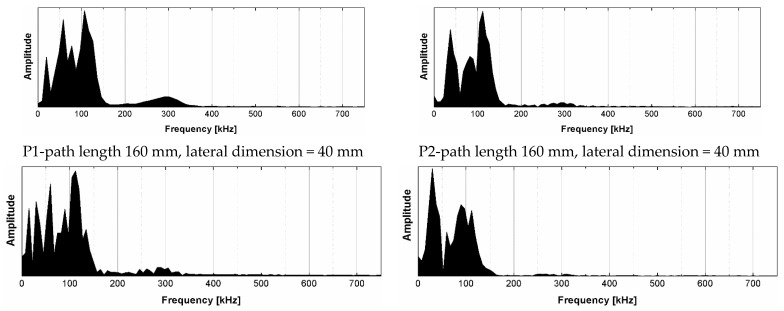
Frequency spectra obtained by fast Fourier transform of signals registered after transmission through epoxy concrete samples with different shape and dimensions.

**Figure 10:**
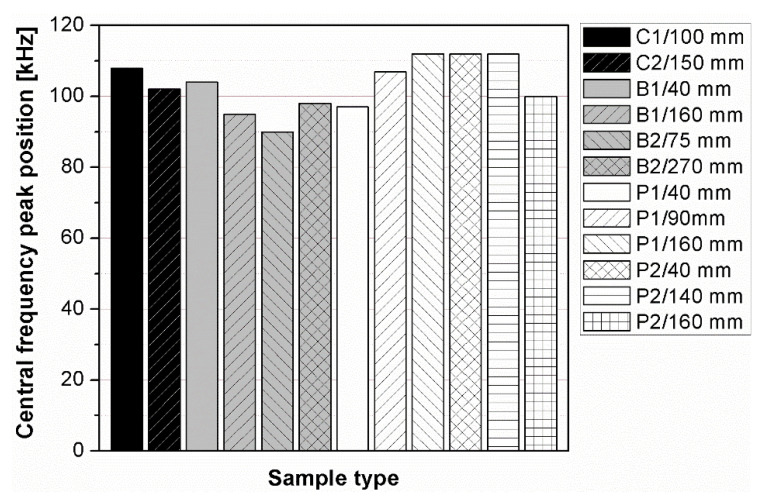
Changes in the position of central frequency peak depending on the sample geometry.

The frequency spectra obtained by fast Fourier transform of the registered ultrasonic signals are presented in Figure 9, while the computed Fourier synchrosqueezed transform of the ultrasonic signals are plotted in the Figure 10.

**Figure 11:**
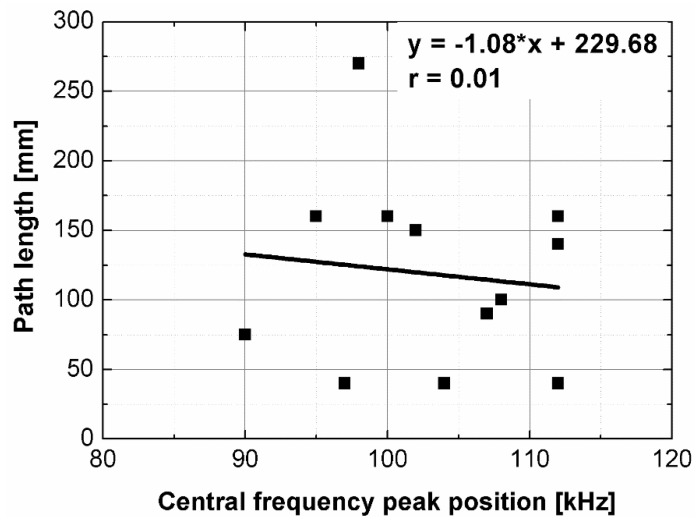
Path length plotted against position of central frequency peak.

**Figure 12:**
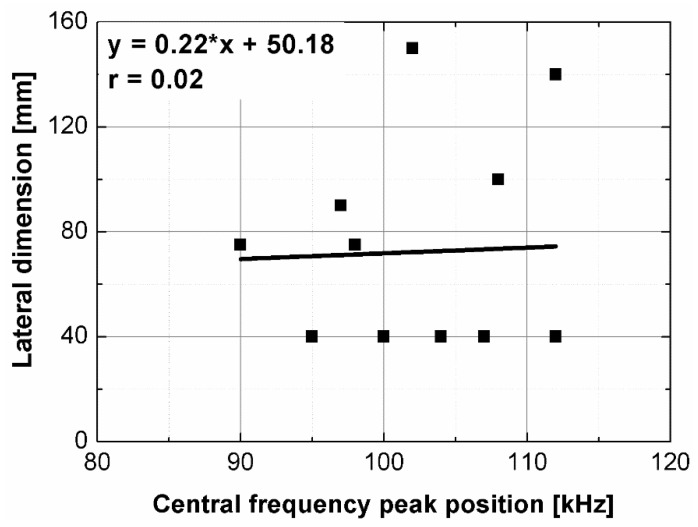
Minimal lateral dimension plotted against position of central frequency peak.

The results of the analysis showed that the signals’ energy is concentrated at the frequency from 0 to 100 kHz. Generally, two kinds of peak may be distinguished in the spectra; first, the peak corresponding to the central frequency of transducers (the nominal value of 100 kHz), which was shifted to the lower or higher frequencies and located between 90–112 kHz (Figure 10); second, at least one peak located near lower frequencies between 30 and 60 kHz. The analysis of changes in the position of central frequency peak (Figure 11 and Figure 12) due to the sample geometry changes revealed that there is no relation between peak position and sample dimensions (path length, minimal lateral dimension), since the values of correlation coefficient |*r*| were below 0.02. It contrasts with the findings of Wiciak P. et al. [35], who noticed that the central frequency peak is shifted to the lower frequencies as the length and diameter of specimens made of cement concrete increases. Both types of peaks demonstrated varied in intensity, and in most cases of analyzed spectra, the peak corresponding to central frequency characterized greater intensity than lower frequency peaks. However, these differences/changes seem not to be associated with sample geometry changes. The differences between spectra may result in wave diffraction on the microstructure constituents.

Compared to other spectra, the spectrum of the signal transmitted along the length of 75 mm × 75 mm × 270 mm beam was the most distinguished; this spectrum showed multiple peaks, located tightly in the range of frequency 30–100 kHz. The reason was probably the greatest path length tested, so the wave attenuation on the microstructure constituents was also the greatest, as the ultrasonic wave travelling through a medium is attenuated due to energy scattering on interfaces with phases having distinct properties, such as voids, cracks, aggregate grains, and reinforcement. Summarizing, the performed analysis of the signal using FFT did not reveal clear influence of sample geometry. The FFT transform does not seem to be sensitive enough to detect potential changes of that kind in signal characteristics.

**Figure 13:**
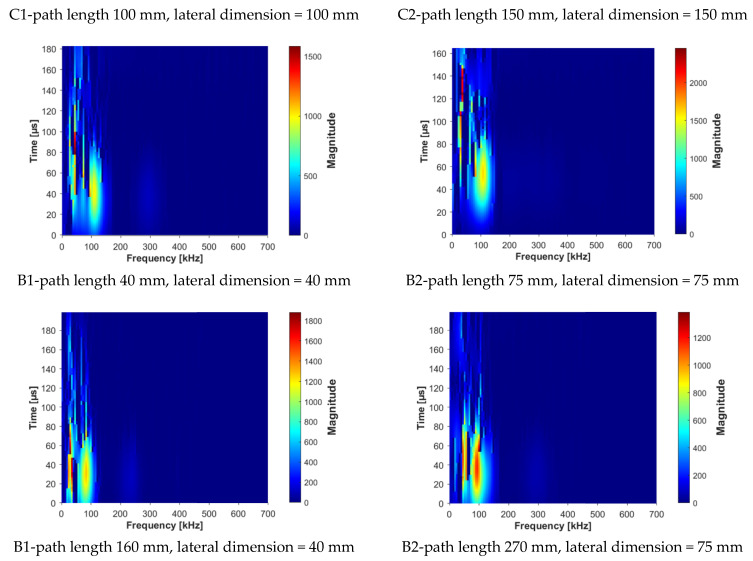

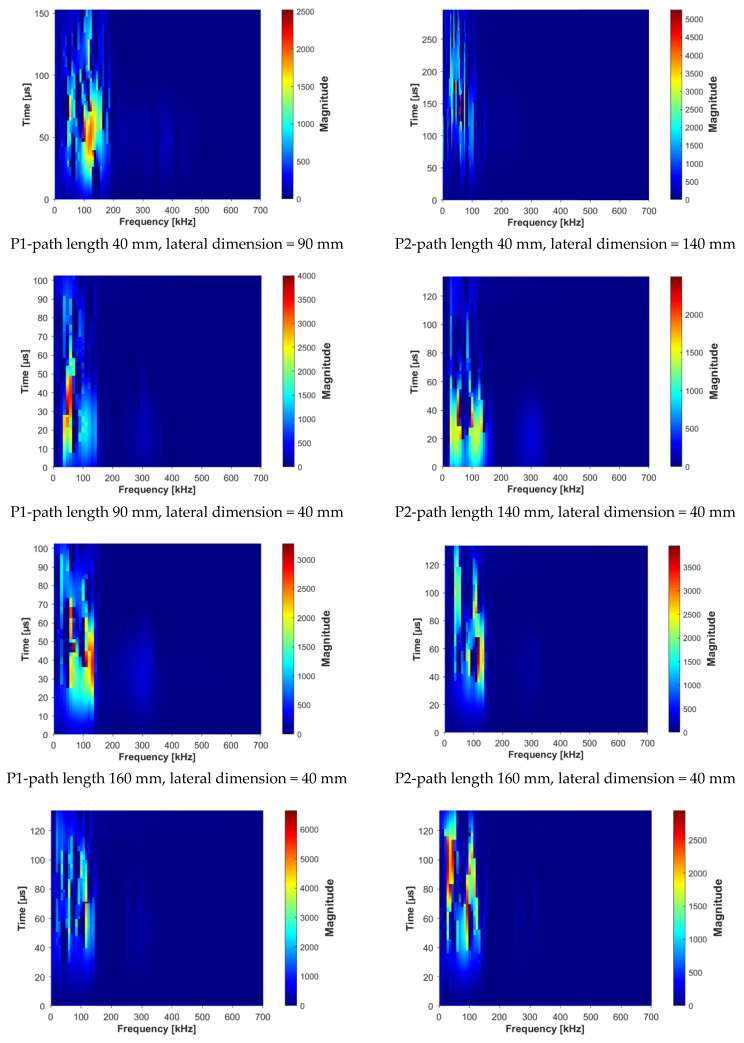
Spectrograms obtained by Fourier synchrosqueezing transform of signals registered after transmission through epoxy concrete samples with different shape and dimensions.

**Figure 14:**
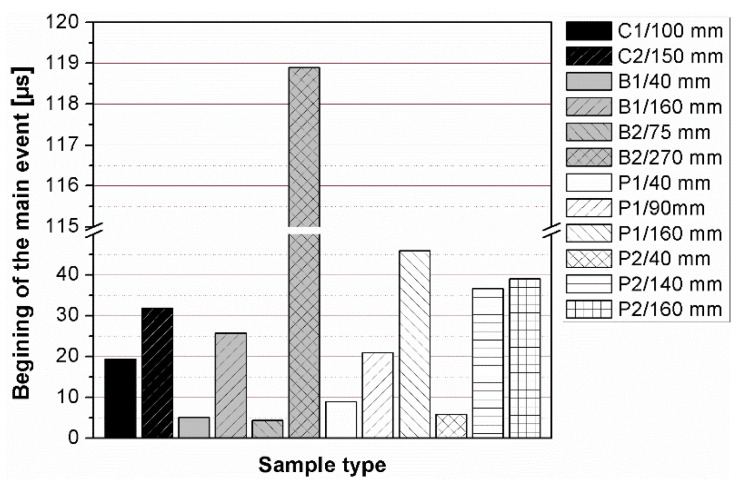
Changes in beginning time of the main event depending on the sample geometry.

**Figure 15:**
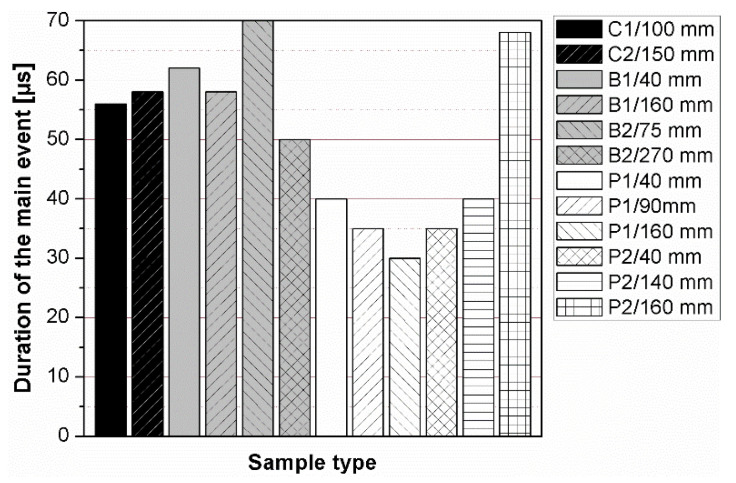
Changes in duration time of the main event depending on the sample geometry.

**Figure 16:**
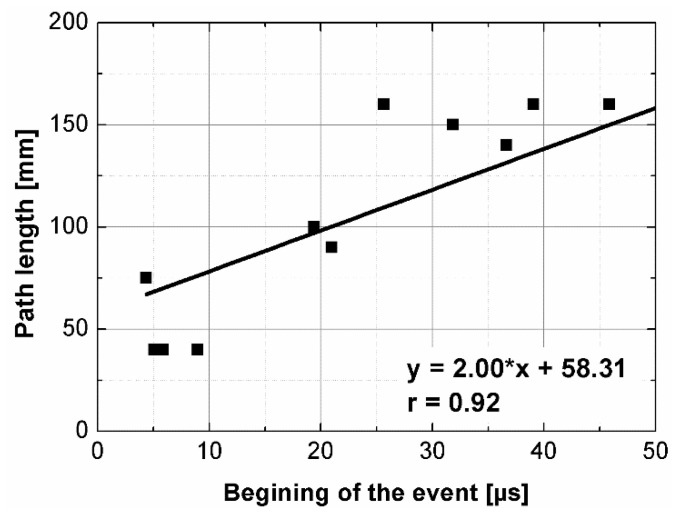
The beginning of the main event plotted against path length.

**Figure 17:**
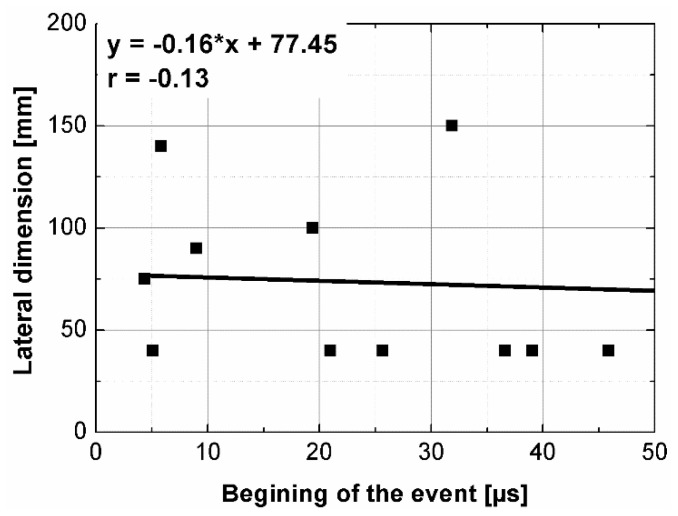
The beginning of the main event plotted against minimal lateral dimension.

**Figure 18:**
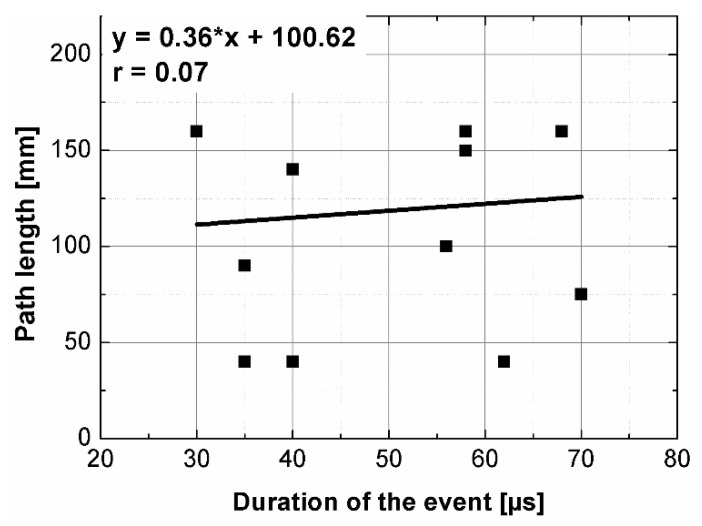
The duration of the main event plotted against path length.

**Figure 19:**
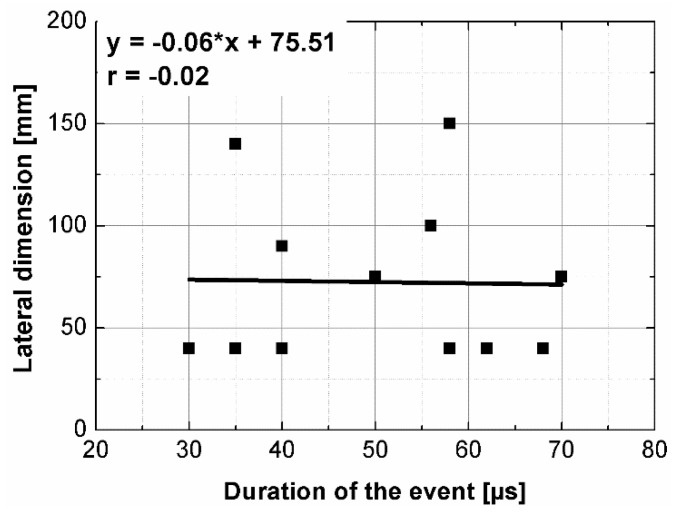
The duration of the main event plotted against minimal lateral dimension.

The instantaneous frequency spectra are shown in the Figure 13. The results of the analysis revealed that the frequency range of all signals was 0 to about 150 kHz. When testing the C1, C2, B1 samples in all planes and B2 sample along shorter side, there was one significant energy area near the main frequency of 100 kHz corresponding to the central frequency of the transducers (main event). On the spectrograms this area was shifted towards frequencies lower or higher than nominal value of 100 kHz, and that was in agreement with the results of FFT analysis. When testing other samples regardless of the testing plane, the energy spectrum appeared to be more dispersed and one significant energy area near the main frequency was not present. It may be clearly seen in the case of the signal registered after transmission through the B2 sample along its length (path length of 270 mm), as a result of the greatest wave dispersion on the microstructure constituents of all samples tested. Another difference between signals are the time of the beginning of the frequency event and the time of its duration (Figure 14 and Figure 15). It was noticed that the greater is the wave propagation path length, the later is the main event. This relation is very strong, as was shown by correlation coefficient |*r*| = 0.92 (Figure 16). The beginning time of the main event was not influenced by the minimal lateral dimension of samples (|*r*| = 0.13, Figure 17), as well as their shape. The changes in the duration time of the main event were not related to sample shape and sizes; these included the path length (|*r*| = 0.07, Figure 18) and lateral dimension (|*r*| = 0.02, Figure 19). Additionally, it could be stressed that the energy of higher frequency bands decays faster than those in low frequency bands.

## 5. Conclusions

The aim of this paper was to analyze how the shape and dimensions of polymer concrete samples may influence the ultrasonic pulse velocity as well as ultrasonic signal in frequency and time-frequency domains. The ultrasonic pulse velocity results were changing with a ratio of 1 ± 0.03 regardless of sample geometry. The correlation analysis showed that neither the path length nor the lateral dimension to the direction of wave propagation are not statistically significant for the UPV variability. However, the general trend of decrease in the UPV with increasing the path length was noticed.

The frequency spectra obtained using fast Fourier transform showed that the signal’s energy is concentrated at the frequency from 0 to 100 kHz. The peak corresponding to transducers’ central frequency of 100 kHz was observed and it was shifted to the lower or higher frequencies and located between 90–112 kHz. The analysis of changes in the position of central frequency peak revealed that there is no relation between peak position and sample shape and sizes. The intensity of peaks observed in the spectra were also changing, but these changes were not associated with the sample geometry changes. Thus, it could be concluded that the geometry of the polymer concrete sample does not influence the ultrasonic signal characteristics in frequency domain.

The analysis of the signal in the time-frequency domain revealed greater differences between signals received for different sample geometries. This approach has the potential to be useful in the analysis of PC or other concrete like composites properties modified with waste products, PC precast elements with different geometry etc., especially when UPV changes are not clear enough. The instantaneous frequency spectra analysis in most cases revealed the presence of a significant energy area near the main frequency of 100 kHz corresponding to the central frequency of the transducers. This area was shifted towards frequencies lower or higher than nominal frequency, and that was in agreement with the results of analysis in the frequency domain. The differences at the beginning of the events and the time of their duration were also noticed. It turned out that beginning of the main frequency event is strongly related to the path length of the wave propagation, but it is not related to the sample shape or lateral dimension. Therefore, it could be stated that spectrograms obtained using Fourier-based synchrosqueezing transform are sensitive to changes in polymer concrete sample sizes, but only changes of dimension parallel to the direction of wave propagation.

## Figures and Tables

**Table 1:** The results of ultrasonic pulse velocity measurements in polymer concrete samples of different geometry [44].

**Pulse** **velocity**	**Sample type**		
	PC (*L/D* = 2)	PC (*L/D* = 3)	PC (*L/b* = 4.5)
	**Sample dimensions**		
	Ø38 mm, *L* = 76 mm	Ø60–65 mm, *L* = 160–190 mm	50 × 50 × 225 mm
*UPV^50^*	3300	3540	3485
*UPV^150^*	3270	3476	3494

Explanations: L/D—length to diameter ratio; L/b—length to width ratio; UPV50—UPV measured at frequency of 50 kHz; UPV150-UPV measured at frequency of 150 kHz.

**Table 2:** Selected physico-chemical and mechanical characteristics of Epidian 601 (CIECH Żywice, Nowa Sarzyna, Podkarpackie voivodeship, Poland) [48].

Characteristics	Value
Epoxy number	0.50–0.55 mol/100 g
Density in 25 °C	1.14–1.17 g/cm^3^
Viscosity in 25 °C	0.7–1.1 Pa·s
Geltime in 20 °C	45 min
Tensile strength	50–60 MPa
Bending strength	80–90 MPa
Barcol hardness	28–33° B

**Table 3:** Shape and dimensions of epoxy concrete samples used in the study.

Cubes	Beams	Plates
C1	B1	P1
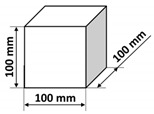	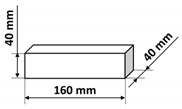	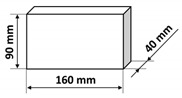
C2	B2	P2
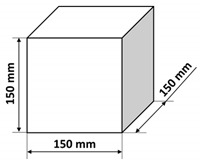	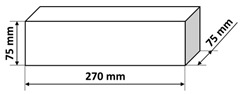	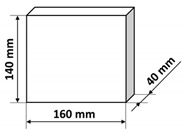

**Table 4:** Mean pulse velocity in epoxy concrete samples calculated for the given path lengths and sample type (all test planes).

Sample Type	Path Length [mm]	Minimal Lateral Dimension [mm]	UPV [m/s]	
			Mean for the Given Path Length	Mean for the Given Sample Type (All Test Planes)
C1	100	100	4155	4155
			(54)	(54)
C2	150	150	3938	3938
			(44)	(44)
B1	40	40	4197	4137
			(59)	
	160	40	4077	(80)
			(42)	
B2	75	75	3909	3877
			(62)	
	270	75	3846	(64)
			(49)	
P1	40	90	3996	4054
			(52)	
	90	40	4159	
			(41)	(77)
	160	40	4006	
			(40)	
P2	40	140	4229	4074
			(42)	
	140	40	4048	
			(45)	(82)
	160	40	3945	
			(38)	
